# Progressive Polycystic Kidney Disease in an Infant Girl With TSC2/PKD1 Contiguous Gene Syndrome

**DOI:** 10.7759/cureus.67800

**Published:** 2024-08-26

**Authors:** Kazuhiko Hashimoto, Takuya Hayashida, Yoshikazu Otsubo, Yo Niida, Sumito Dateki

**Affiliations:** 1 Department of Pediatrics, Sasebo City General Hospital, Sasebo, JPN; 2 Division of Genomic Medicine, Department of Advanced Medicine, Medical Research Institute, Kanazawa Medical University, Uchinada, JPN; 3 Center for Clinical Genomics, Kanazawa Medical University Hospital, Uchinada, JPN; 4 Department of Pediatrics, Nagasaki University Graduate School of Biomedical Sciences, Nagasaki, JPN

**Keywords:** tuberous sclerosis complex, renal size volumetry, polycystic kidney disease, hypertension, contiguous gene syndrome

## Abstract

TSC2/PKD1 contiguous gene syndrome is caused by deletions involving the TSC2 and PKD1 genes that lead to tuberous sclerosis complex and autosomal dominant polycystic kidney disease. It is characterized by early-onset severe cystic kidney disease with progressive enlargement of the kidneys and the cysts. As it can lead to early hypertension and an accelerated decline of kidney function, early genetic testing is needed for early diagnosis of this syndrome, and more frequent imaging-based examinations are necessary to assess disease progression and determine appropriate management. We report the case of an infant girl with TSC2/PKD1 contiguous gene syndrome who presented with epileptic seizures. Brain magnetic resonance imaging (MRI) revealed subependymal nodules and cortical tubers, and abdominal MRI revealed polycystic kidney lesions and enlargement of both kidneys. TSC2/PKD1 contiguous gene syndrome was suspected from her radiological features, and we confirmed the presence of a deletion in the girl’s genome, which included the TSC2 and PKD1 genes, via microarray analysis. Thereafter, we evaluated the change in kidney size via repeated abdominal MRI. The polycystic kidney lesions enlarged, and the patient developed hypertension in early childhood, for which we administered an angiotensin-converting enzyme inhibitor. We emphasize the importance of evaluation with longitudinal abdominal imaging because renal cysts tend to enlarge rapidly and induce hypertension, as demonstrated in our case.

## Introduction

Tuberous sclerosis complex (TSC) is an autosomal dominant multisystem disorder characterized by hamartomas in multiple organs, including the brain, skin, heart, lungs, and kidneys [1]. TSC is caused by mutations in either TSC1 or TSC2, which encode hamartin and tuberin, respectively [2]. Mutations of the polycystic kidney disease type 1 (PKD1) gene, which lies adjacent to TSC2 on chromosome 16p13.3, are responsible for autosomal dominant polycystic kidney disease (ADPKD) [3]. ADPKD is characterized by progressive bilateral kidney cysts, and most individuals with PKD1 mutations have kidney failure by age 70 (the mean age of onset is 54.3 years) [3]. Large deletions encompassing both the TSC2 and PKD1 genes result in TSC2/PKD1 contiguous gene syndrome and occur in 2%-5% of patients with TSC [4].

The characteristic phenotype of patients with TSC2/PKD1 contiguous gene syndrome is the presence of early-onset polycystic kidney lesions, which tend to grow more rapidly than those in patients with classical TSC, resulting in kidney failure in the teenage years [5]. Few case reports of TSC2/PKD1 contiguous gene syndrome have been published, and physicians should be made aware of its physical and radiographic features.

Herein, we report the case of an infant girl with TSC2/PKD1 contiguous gene syndrome followed by sequential kidney lesions upon abdominal magnetic resonance imaging (MRI).

## Case presentation

The patient was born at term as the first child of healthy, non-consanguineous Japanese parents with an unremarkable family history. At 10 months of age, she developed sudden behavioral arrest and ankylosis of the upper limbs. Electroencephalography (EEG) revealed no abnormalities; therefore, she was observed without any anti-epileptic medications. Similar symptoms recurred at 1 year 7 months of age, and she was admitted to our hospital for further examination at 1 year 9 months of age.

**Figure 1:**
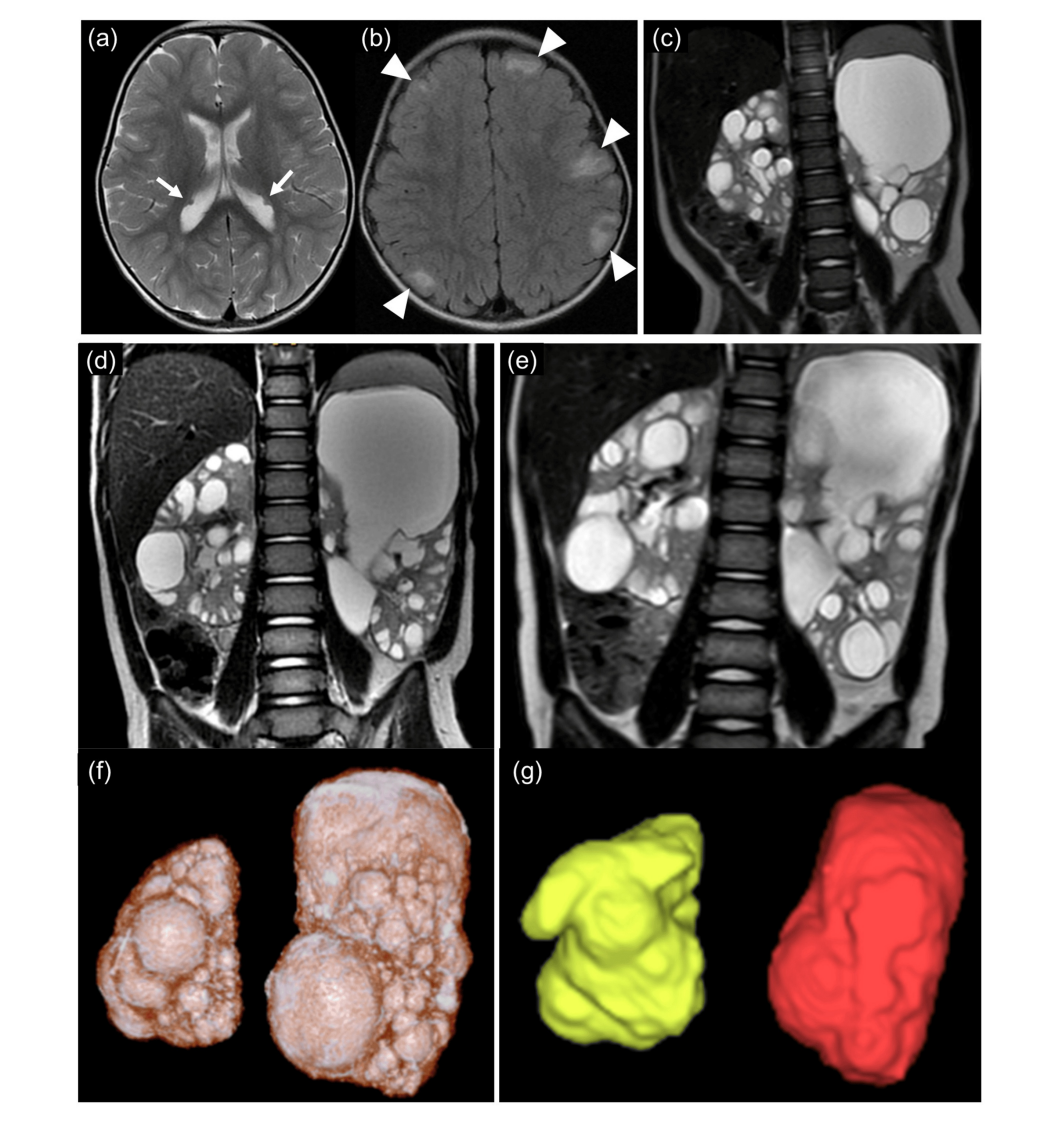
Imaging of an infant with TSC2/PKD1 contiguous gene syndrome. (a) Axial T2-weighted magnetic resonance imaging (MRI) of the head at 1 year and 9 months of age revealing hypointense subependymal nodules in the posterior of the lateral ventricles (arrows). (b) Axial fluid-attenuated inversion recovery image revealing multiple cortical tubers (arrowheads). (c) Coronal T2-weighted abdominal MRI at 1 year and 10 months of age revealing asymmetrically enlarged kidneys with polycystic lesions of various sizes. (d) Coronal T2-weighted abdominal MRI at 2 years 7 months of age and (e) at 3 years 9 months of age. (f) The kidney size volumetry; right: 194 ml, left: 546 ml at 2 years 7 months of age and (g); right: 287 ml, left: 559 ml at 3 years 9 months of age. These revealed gradual enlargement of the kidneys and advancement of polycystic kidney disease.

Upon admission, the patient had a hypomelanotic macule on the abdomen. She did not present neurological abnormalities or developmental delays. Long-term video-EEG monitoring revealed interictal discharges in the left frontal lobe during sleep. Brain MRI revealed subependymal nodules and cortical tubers (Figures 1a, 1b). Abdominal MRI revealed enlargement of both kidneys and polycystic renal lesions (Figure 1c). The kidney volume was 562 ml (right: 130 ml; left: 432 ml). We suspected TSC2/PKD1 contiguous gene syndrome based on these symptoms and features.

Reexamination with abdominal MRI at 2 years 7 months and 3 years 9 months of age revealed gradual enlargement of the cysts in both kidneys (Figures 1d, 1e). The kidney size volumetry measured via these two MRIs were 740 ml (right: 194 ml; left: 546 ml) and 846 ml (right: 287 ml; left: 559 ml), respectively (Figures 1f, 1g). Periodic brain MRI revealed no changes in her intracranial lesions and no aneurysm, which is a reported complication of this syndrome [2]. The patient presented with hypertension at 2 years 8 months of age and required treatment with an angiotensin-converting enzyme inhibitor (ACE-I), which normalized her blood pressure by her latest visit at 4 years of age. However, she still experienced behavior arrest seizures on a weekly basis despite treatment with valproate and levetiracetam.

**Figure 2:**
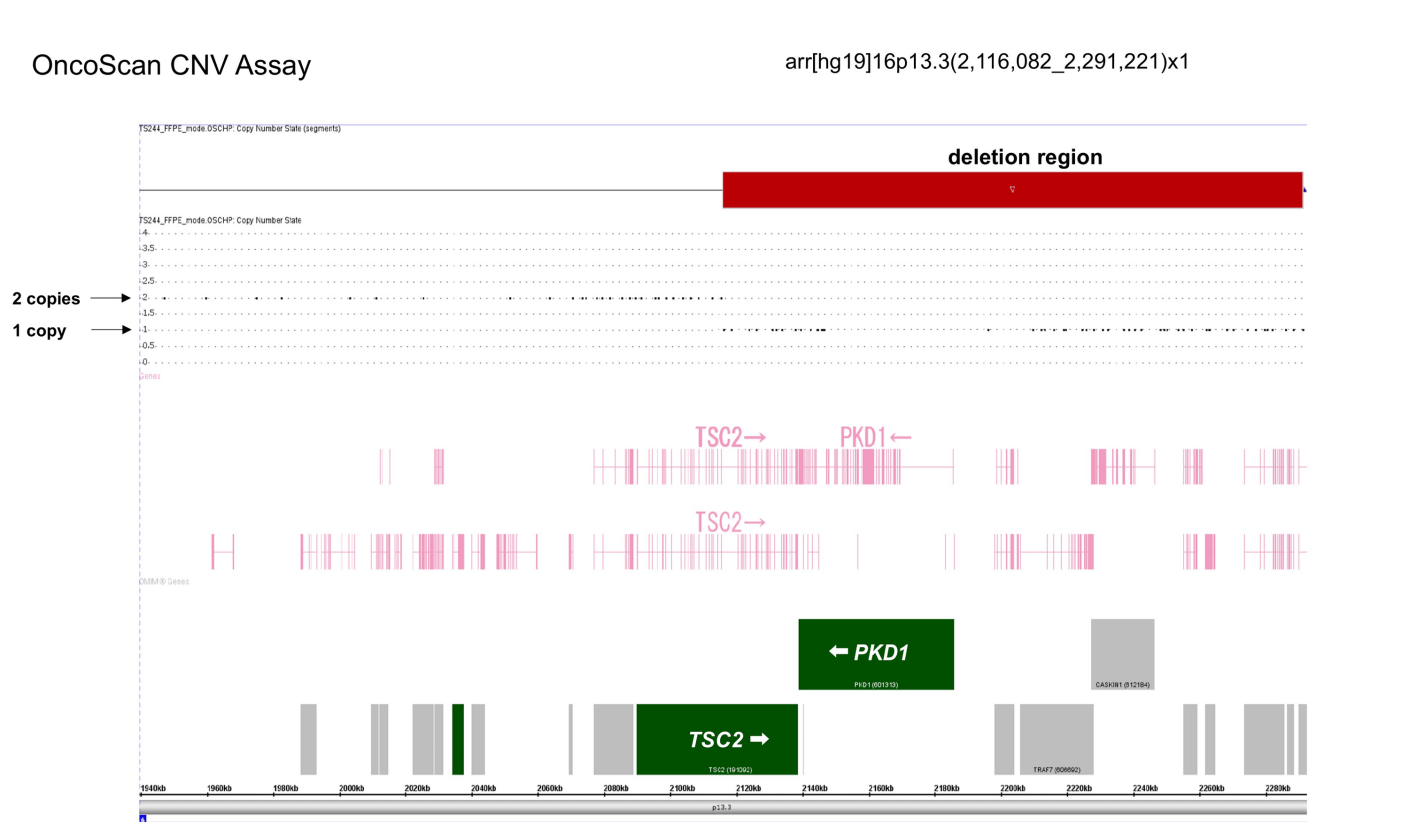
Genetic analysis of the TSC2/PKD1 region. Microarray analysis (OncoScan CNV Assay; Thermo Fisher Scientific, Waltham, MA, USA) revealed only one copy region (red bar) encompassing TSC2 and PKD1 genes. Therefore, we confirmed a TSC2/PKD1 contiguous gene syndrome diagnosis based on genetic analysis. CNV, copy number variation.

We performed a genetic analysis of the TSC1 and TSC2 genes via long-range polymerase chain reaction (PCR)-based next-generation sequencing, which revealed no point mutations, deletions/duplications, mosaicism, or splicing mutations. We also performed microarray analysis (OncoScan CNV (Copy Number Variation) Assay; Thermo Fisher Scientific, Waltham, MA, USA), which revealed a 175-kb deletion encompassing TSC2 and PKD1 (arr(hg19)16p13.3(2,116,082_2,291,221)x1, Figure 2). Therefore, the patient was diagnosed with TSC2/PKD1 contiguous gene syndrome. Informed consent was obtained from the patient’s parents for publication of this report.

## Discussion

We reported the case of an infant girl with TSC2/PKD1 contiguous gene syndrome manifesting as epilepsy and early-onset polycystic kidney lesions. We also described sequential abdominal MRI which revealed progression and enlarging of both polycystic kidneys.

Kidney cysts are one of the most common kidney manifestations of TSC, with a prevalence of 16%-32%. Such cysts most frequently occur in the second decade of life [6,7]; however, those of patients with TSC2/PKD1 contiguous gene syndrome have an early onset and enlarge earlier than those of patients with typical TSC [4]. Sampson et al. reported that the median age at onset of kidney cysts in 17 patients was 6 months (range: 1 month to 10 years), and three of those patients required replacement therapy for kidney failure before 30 years of age [8]. In our case, the onset of the cysts was uncertain, but we assume that they appeared in infancy at the latest because our patient had already developed polycystic kidney disease by the initial MRI. Since no clinical markers have been identified in TSC2/PKD1 contiguous gene syndrome, clinicians should consider the possibility of this syndrome when they detect early-onset polycystic kidney lesions in patients with typical manifestations of TSC.

Grantham et al. assessed changes in kidney volume over a three-year period among 214 patients with ADPKD by using MRI, which revealed that total kidney and total cyst volume increased annually in most patients, with a rate of increase of 5.27%±3.92% per year [9]. In addition, Kistler et al. revealed that the total kidney volume (TKV) change was greater in patients under 30 years of age than that in patients over 30, which means that the rate of TKV change is inversely correlated with age [10]. In our patient, the kidney volume increased by approximately 1.5 times in two years (from 562 ml to 846 ml), a higher rate of increase in TKV than that in previous reports.

The sequential abdominal MRI revealed progressive enlargement of the kidneys and polycystic lesions, which resulted in hypertension in our patient. In the study by Gabow et al., increased kidney volume was due to increased cyst volume, and the early onset of hypertension in patients with ADPKD might have been related to bilateral kidney ischemia caused by changes in kidney vasculature [11]. Early detection and treatment of hypertension are important for the management of PKD because uncontrolled hypertension increases the risk of various diseases (e.g., cardiovascular disease and decline of kidney function) [3]. No antihypertensive drugs have been recommended for PKD; however, ACE-I and angiotensin receptor blockers can have renoprotective properties beyond blood pressure control and have low side effects [3]. An analysis of 142 patients with ADPKD in eight randomized clinical trials revealed that ACE-I provided excellent blood pressure control and lowered the urine protein concentration in patients with PKD [12]. ACE-I was similarly effective in our case.

Tolvaptan and vasopressin V2-receptor antagonists are the therapeutic agents for ADPKD in adults; they slowed down the increase in TKV and the decline in kidney function over a three-year period in one study [13,14]. Tolvaptan also slowed the decline of the estimated GFR over a one-year period in patients with later-stage ADPKD [15]. However, it is not indicated for use in children, and few reports of its use in children have been published. Mekahli et al. reported the first phase 3, interventional, clinical trial of tolvaptan in children and adolescents with ADPKD, a cohort study of 66 patients aged 12-17 years and 25 patients aged 4-11 years [16]. Although not statistically significant, participants aged 12-17 had a lower rate of kidney volume growth with tolvaptan (2.6%) than with placebo (5.8%) over the course of 12 months. They also described children in the tolvaptan group had a significantly higher incidence of aquaretic side effects such as thirst, polydipsia, and polyuria (65%) compared with children in the placebo group (16%), however, only one participant in the tolvaptan group discontinued treatment and none of the tolvaptan-treated participants experienced liver toxicity [16]. Muroga et al. reported the case of a child with TSC2/PKD1 contiguous gene syndrome successfully treated with tolvaptan for rapidly enlarging renal cysts [17]. The patient had no apparent adverse events and the MRI performed 12 months after treatment initiation showed that renal cyst enlargement was suppressed [17]. Based on these results, this drug may be effective for pediatric patients with TSC2/PKD1 contiguous gene syndrome, however, tolvaptan's safety for long-term use across the pediatric age spectrum is unclear. Although earlier initiation of tolvaptan could confer benefits for long-term kidney survival, children are especially vulnerable to the risks of profound aquaresis, treatment of children must account for careful assessment of risks of side effects [18]. Therefore, the use of tolvaptan in our case should be considered the balance of disease progression and medication side effects.

## Conclusions

In this report, we highlight the importance of assessment with abdominal imaging to detect kidney disease when examining patients with tuberous sclerosis complex. Patients with TSC2/PKD1 contiguous gene syndrome develop early-onset polycystic kidney lesions, which tend to grow more rapidly than those in classical autosomal dominant polycystic kidney disease. In addition, early intervention for kidney complications such as hypertension is also important to prevent kidney failure, so genetic testing should be performed at the time of suspicion of this syndrome. Early initiation of tolvaptan may prevent the progression of the disease in pediatric patients but the necessity of this drug should be considered carefully for risks of side effects.
